# Silencing lncRNA Lfar1 alleviates the classical activation and pyoptosis of macrophage in hepatic fibrosis

**DOI:** 10.1038/s41419-020-2323-5

**Published:** 2020-02-18

**Authors:** Kun Zhang, Zhemin Shi, Mengxia Zhang, Xueyi Dong, Lina Zheng, Guantong Li, Xiaohui Han, Zhi Yao, Tao Han, Wei Hong

**Affiliations:** 1 Department of Histology and Embryology, Tianjin Key Laboratory of Cellular and Molecular Immunology, Key Laboratory of Immune Microenvironment and Disease of Ministry of Education, School of Basic Medical Sciences, Tianjin Medical University, Tianjin, China; 2 Department of Pathology, School of Basic Medical Sciences, Tianjin Medical University, Tianjin, China; 3 The Third Central Clinical College of Tianjin Medical University, Department of Hepatology and Gastroenterology, Tianjin Third Central Hospital affiliated to Nankai University, Tianjin Key Laboratory of Artificial Cells, Artificial Cell Engineering Technology Research Center of Public Health Ministry, Tianjin, China; 4 Department of Immunology, Tianjin Key Laboratory of Cellular and Molecular Immunology, Key Laboratory of Immune Microenvironment and Disease of Ministry of Education, School of Basic Medical Sciences, Tianjin Medical University, Tianjin, China

**Keywords:** Cell death, Long non-coding RNAs, Liver fibrosis

## Abstract

Hepatic fibrosis is a common pathological consequence of a sustained wound healing response to continuous liver injury, characterized by increased production and accumulation of extracellular matrix. If unresolved, the fibrotic process results in organ failure, and eventually death after the development of cirrhosis. It has been suggested that macrophages play central role in the progression of hepatic fibrosis, which is related to inflammation and pyroptosis, a novel programmed and proinflammatory cell death. However, it remains far less clear if, or how, lncRNAs regulates the activation and pyroptosis of macrophage in hepatic fibrosis. In the present study, we demonstrated that the liver-enriched lncRNA Lfar1, which has been reported to promote hepatic fibrosis through inducing hepatic stellate cells activation and hepatocytes apoptosis, was dysregulated during proinflammatory M1 activation and pyroptosis of macrophage. Our study revealed that silencing lnc-Lfar1 by a lentivirus-shRNA alleviated CCl_4_- and BDL-induced proinflammatory M1 macrophage activation and NLRP3 inflammasome-mediated pyroptosis. Furthermore, the in vitro experiments demonstrated that lnc-Lfar1 knockdown significantly suppressed LPS- and IFN-γ-induced proinflammatory activation of macrophages, and inhibited LPS/ATP- and LPS/Nigericin-induced NLRP3 inflammasome-mediated pyroptosis. Mechanistically, lnc-Lfar1 regulated LPS- and IFN-γ-induced proinflammatory activation of macrophages through the NF-ĸB pathway. All these data supported our conclusion that lnc-Lfar1 plays a vital role in controlling the activation and pyroptosis of macrophage, thus providing a possible therapeutic target against inflammation-related disorders including hepatic fibrosis.

## Introduction

Chronic hepatic inflammation is tightly linked to the pathogenesis of fibrosis, and hepatic fibrosis is a sequel of a sustained wound-healing process in response to a continuous liver injury, characterized by increased production and accumulation of extracellular matrix^1^. Several studies have emphasized the central role of macrophages for the progression of liver inflammation and fibrosis^2,3^. During recent years, it has become clear that hepatic macrophages (HMs) are heterogeneous cell populations, consisting of liver-resident macrophage, traditionally termed Kupffer cells (KCs), and monocyte-derived macrophages (MoMFs) that are recruited from the circulation to the liver upon acute or chronic liver injury^4–6^. Macrophages can be roughly classified into classically activated (M1) macrophages induced by T helper type 1 (Th1) signals, including interferon-gamma (IFN-γ) or lipopolysaccharides (LPS), and alternatively activated (M2) macrophages activated by Th2 signals, such as interleukin-4 (IL-4), IL-10, IL-13 and parasites. Although M2 macrophages were shown to act profibrotic in pulmonary and hepatic fibrosis, it is well accepted that M1 cells secrete large amounts of proinflammatory cytokines, including IL-1β, IL-6, monocyte chemoattractant protein-1 (MCP-1) and tumor necrosis factor-α (TNF-α), which contribute to hepatocyte apoptosis, promote inflammatory cell recruitment, activate hepatic stellate cells (HSCs) and perpetuate fibrosis^4–6^. Thus, understanding of macrophage polarization and biology during hepatic fibrosis might result in potential antifibrotic therapies.

Pyroptosis is a novel programmed and proinflammatory cell death, which is initiated by inflammasomes and executed by Gasdermin D (GSDMD), leading to pore formation in the plasma membrane, fluid influx, cell swelling, rapid plasma membrane rupture and massive release of lactate dehydrogenase (LDH) and the proinflammatory cytokines such as IL-1β and IL-18^7,8^. Inflammasomes are a group of multimeric protein complexes usually composed of the activated NOD-like receptor (NLR), the adapter apoptosis-associated speck-like protein containing CARD (ASC) and the effector pro-caspase 1. The NLRP3 inflammasome is currently the most well-characterized inflammasome and is assembled in the cytosol in response to pathogen-associated molecular patterns (PAMPs) such as LPS and flagellin derived from the gut, or damage-associated molecular patterns (DAMPs) derived from damaged hepatocyte, adenosine triphosphate (ATP), DNA fragments and reactive oxygen species (ROS). Upon activation, the N-terminal pyrin segment (PYD) of NLRP3 serves as a scaffold to induce the oligomerization of ASC and recruits pro-caspase-1. Then, the oligomerization of pro-caspase1 proteins facilitates its auto-processing into active caspase1 or cleaved caspase1, which cleaves pro-IL-1β and pro-IL-18 into biologically active cytokines IL-1β and IL-18, respectively^2,7,8^. In addition to processing proinflammatory cytokines, active caspase-1 also cleaves the Gasdermin-D into a 22-kDa C-terminal fragment and a 31-kDa N-terminal fragment (GSDMD-N), which forms the membrane pores to induce mature IL-1β and IL-18 release^8^. Growing evidences suggest that NLRP3 inflammasome and pyroptosis play an important role in liver inflammation and fibrosis^7,9–11^. It has been reported that NLRP3 hyperactivation-initiated pyroptosis resulted in more severe liver inflammation and fibrosis, while NLRP3 deficiency alleviates carbon tetrachloride (CCl_4_)-induced liver fibrosis^10^. Similarly, studies using mice genetically deficient of caspase and ASC have also demonstrated a protective effect on fibrosis development^2^. Moreover, hepatic nutritional fibrosis was strongly attenuated in GSDMD^−/−^ mice after 8 weeks of MCD induction^11^. Therefore, the inhibition of NLRP3 inflammasome and GSDMD is a new strategy for the prevention and treatment of liver inflammation and fibrosis.

The majority of transcripts transcribed from human or mouse genome are non-coding RNAs, such as microRNA (miRNA) and long non-coding RNA (lncRNA), which are a group of transcripts longer than 200 nucleotides but do not encode protein^12^. Emerging evidences have demonstrated that lncRNAs play critical roles in a variety of physiological and pathological processes, including cell proliferation, cell cycle progression, differentiation, apoptosis and inflammation, through regulating gene expression at the epigenetic, transcriptional and posttranscriptional levels^12–16^. Recently, several studies have reported that lncRNAs are involved in the regulation of macrophage activation and pyroptosis^15,17^. For instance, Zhang et al.^17^ demonstrated that lncRNA nuclear enriched abundant transcript 1 (Neat1) is released from paraspeckles and translocated to the cytoplasm in response to various inflammasome-activating signals, then, lncRNA Neat1 binds to pro-caspase-1 and facilitates the assembly of inflammasomes, cytokine production, and pyroptotic cell death. Yang et al.^15^ reported that lncRNA Kcnq1ot1 acts as a competitive endogenous RNA (ceRNA) for miR-214-3p to regulate the expression of caspase-1, and Kcnq1ot1 silencing promotes GSDMD cleavage and the secretion of IL-1β, thus ameliorating pyroptosis and fibrosis in diabetic cardiomyopathy. Moreover, lncRNA metastasis associated lung adenocarcinoma transcript 1 (MALAT1) was recently identified as a pyroptosis-related lncRNA, which promotes high glucose-induced pyroptosis of endothelial cells partly by affecting NLRP3 expression through competitively binding miR-22^18^. However, the effect and mechanism of lncRNAs on pyroptosis and activation of macrophage in hepatic fibrosis remains inadequately investigated.

In the present study, we demonstrated that the liver-enriched lncRNA Lfar1, which has been reported to promote hepatic fibrosis through inducing HSCs activation and hepatocytes apoptosis, was dysregulated during proinflammatory M1 activation and pyroptosis of macrophage. Our study revealed that silencing lnc-Lfar1 alleviated CCl_4_- and BDL-induced proinflammatory M1 macrophage activation and NLRP3 inflammasome-mediated pyroptosis. Furthermore, in vitro experiments demonstrated that lnc-Lfar1 knockdown significantly suppressed LPS- and IFN-γ-induced proinflammatory activation of macrophages, and inhibited LPS/ATP- and LPS/Nigericin-induced NLRP3 inflammasome-mediated pyroptosis. Mechanistically, lnc-Lfar1 regulated LPS- and IFN-γ-induced proinflammatory activation of macrophages through the NF-ĸB pathway.

## Results

### Dysregulation of lnc-Lfar1 in macrophage activation and hepatic fibrosis model

**Fig. 1:**
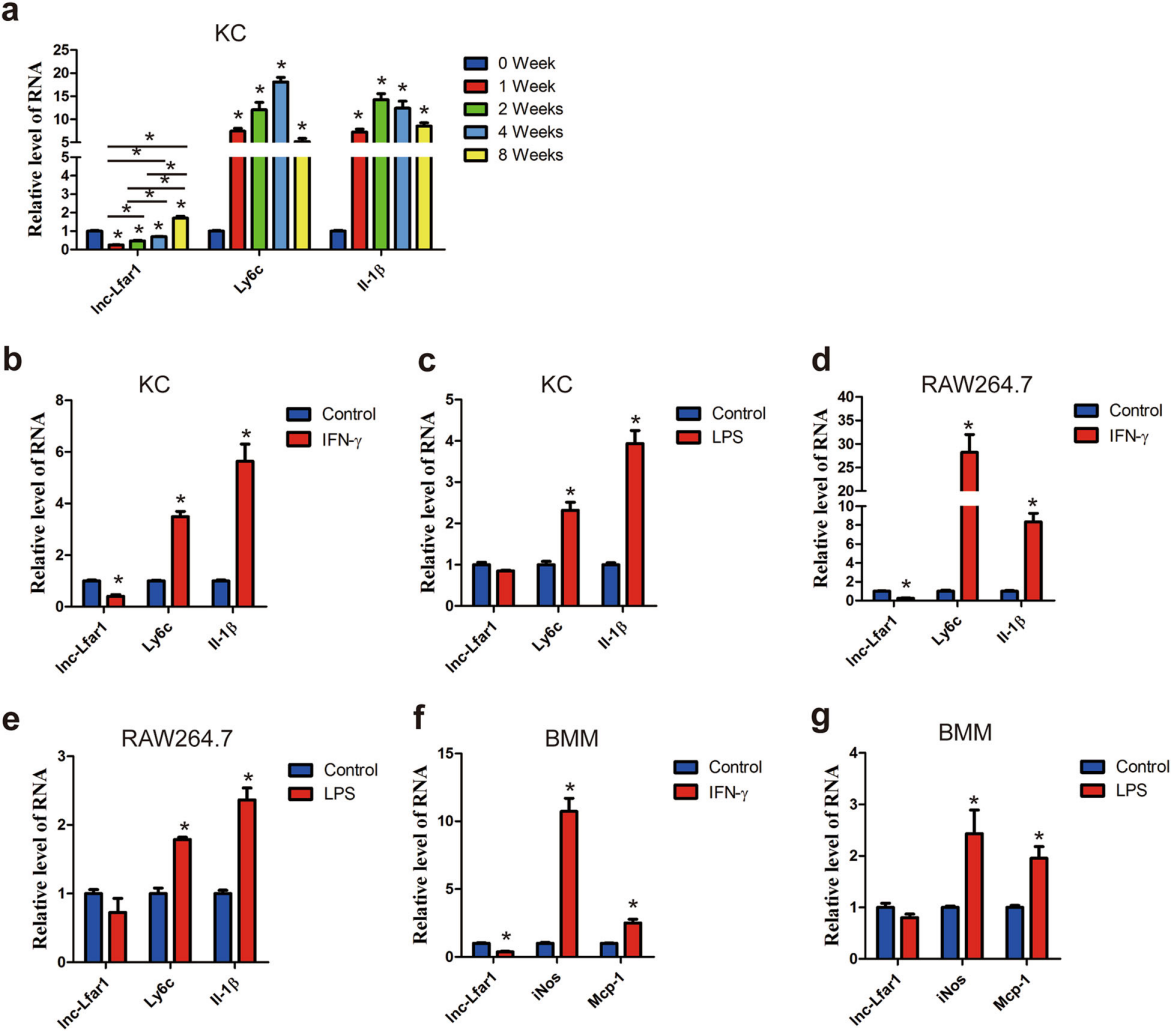
Dysregulation of lnc-Lfar1 in macrophage activation. **a** qRT-PCR analysis of the expression of *lnc-Lfar1, Ly6c* and *Il-1β* in KCs isolated from mice treated with CCl_4_ for 0, 1, 2, 4 or 8 weeks. **b–g** KCs, RAW264.7 and BMMs were stimulated with 20 ng/ml IFN-γ or 100 ng/ml LPS for 24 h and the expression of *lnc-Lfar1* and positive control genes (*Ly6c, Il-1β, iNos* or *Mcp-1*) was determined by qRT-PCR. *p < 0.05 vs control.

Our previous study demonstrated that the liver-enriched lncRNA Lfar1 promoted HSCs activation and subsequently led to liver fibrosis^19^. Moreover, lnc-Lfar1 showed a comparable expression level in HSCs and KCs, and the microarray analysis revealed that lnc-Lfar1 silencing affected a list of genes associated with immune response and responded to IFN-γ in CCl_4_-induced liver fibrosis. Thus, we speculated whether lnc-Lfar1 played a vital role in macrophage activation. To test this hypothesis, we initially examined the expression of lnc-Lfar1 in KCs of mice treated with CCl_4_ for 0, 1, 2, 4 or 8 weeks. KCs were identified by confocal as F4/80^+^/α-SMA^−^/VEGFR2^−^ cells (Supplementary Fig. 1). Interestingly, the level of lnc-Lfar1 was decreased drastically at 1 week after CCl_4_ injection, while gradually increased with persisting CCl_4_ injection (Fig. 1a). To further examine the expression of lnc-Lfar1 in vitro, we treated normal mouse primary KCs with a panel of macrophage-activating cytokines, including IFN-γ, LPS, IL-4 and IL-10. We observed that 20 ng/ml IFN-γ-activated macrophages strongly inhibited lnc-Lfar1 expression (Fig. 1b), while 100 ng/ml LPS, 20 ng/ml IL-4 and 20 ng/ml IL-10 did not regulate its expression in KCs (Fig. 1c and Supplementary Fig. 2a, b). In addition, the expression of lnc-Lfar1 was also detected in murine immortalized macrophages RAW264.7 cells and mouse bone marrow-derived macrophages (BMMs), after IFN-γ, LPS, IL-4 and IL-10 treatment. Consistently, lnc-Lfar1 level decreased in IFN-γ-activated RAW264.7 cells and BMMs, while did not change in IL-4- and IL-10-activated RAW264.7 cells and BMMs (Fig. 1d–g and Supplementary Fig. 2c–f). Taken together, our results suggest that lnc-Lfar1 is downregulated in IFN-γ-induced proinflammatory activation of macrophages.

### Silencing lnc-Lfar1 alleviates CCl_4_- and BDL-induced proinflammatory activation of macrophages

**Fig. 2:**
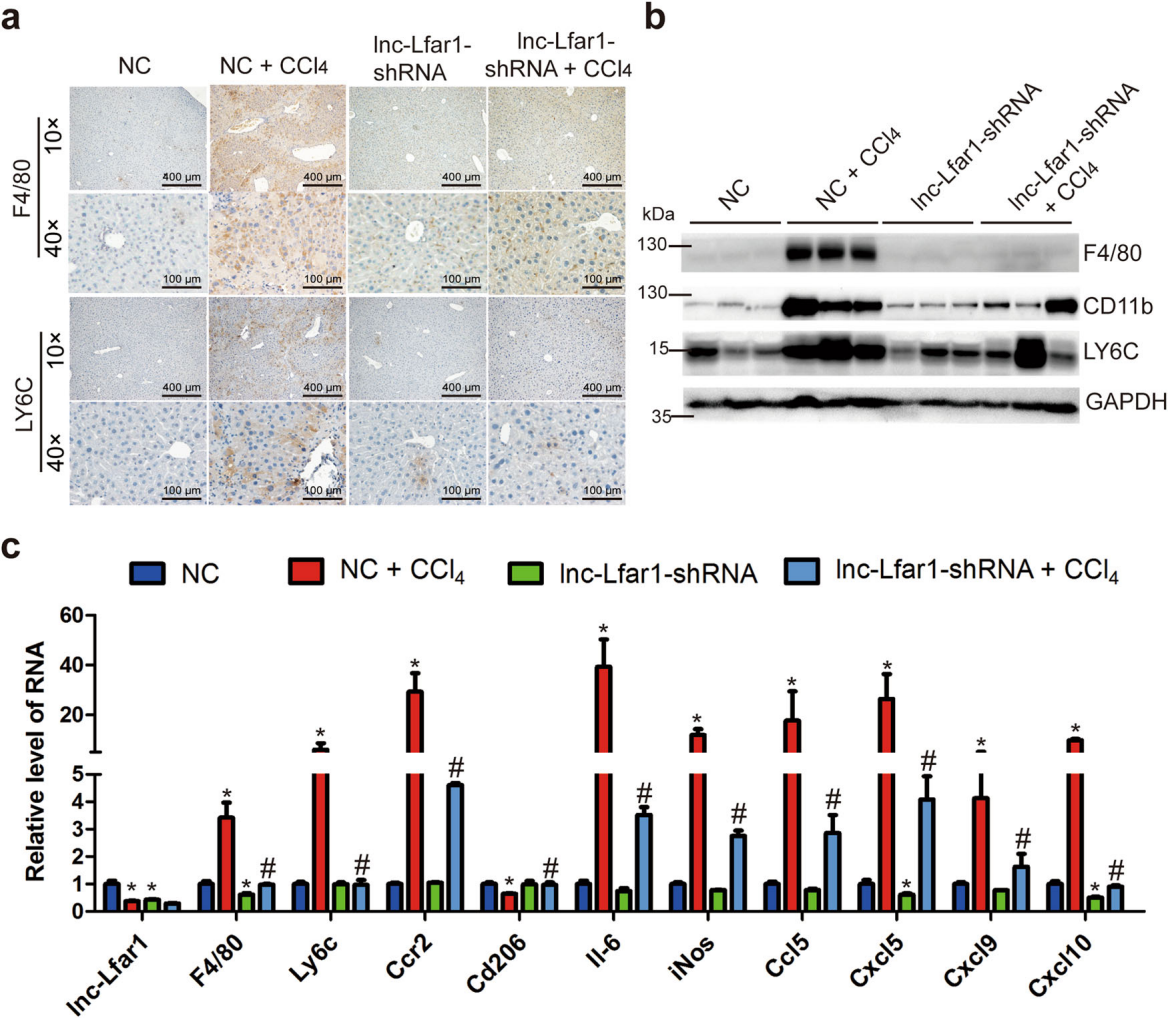
Silencing lnc-Lfar1 alleviates CCl_4_-induced proinflammatory activation of macrophages. Mice were treated with oil in combination with injection of lenti-NC (NC, *n* = 10), CCl_4_ in combination with injection of lenti-NC (NC + CCl_4_, *n* = 10), oil in combination with injection of lenti-lnc-Lfar1-shRNA (lnc-Lfar1-shRNA, *n* = 10), and CCl_4_ in combination with injection of lenti-lnc-Lfar1-shRNA (lnc-Lfar1-shRNA + CCl_4_, *n* = 10). **a** Immunohistochemistry analysis was performed to detect the expression of F4/80 and LY6C; scale bar = 400 μm for 10 × and 100 μm for 40×. **b** The protein level of F4/80, CD11b and LY6C was determined by western blot. GAPDH was used as an internal control. **c** The mRNA level of *F4/80, Ly6c*, *Ccr2*, *Cd20, Il-6, iNos, Ccl5, Cxcl5, Cxcl9* and *Cxcl10* was determined by qRT-PCR. *p < 0.05 vs NC, ^#^*p* < 0.05 vs NC + CCl_4_.

To investigate the potential function of lnc-Lfar1 in the proinflammatory response of M1 macrophages in vivo, lenti-lnc-Lfar1-shRNA or lenti-NC was intravenously injected into CCl_4_- and BDL-treated mice via the tail vein. Immunohistochemistry and immunoblotting analysis indicated that the expression of F4/80, CD11b and LY6C, which is a marker of proinflammatory macrophage activation, was markedly increased in the CCl_4_ group mice infected with NC (NC + CCl_4_), while was decreased after treatment with lnc-Lfar1-shRNA (Fig. 2a, b). Moreover, the mRNA level of the M1 surface markers *F4/80, Ly6c* and *Ccr2*, the proinflammatory cytokines including *Il-6, iNos, Ccl5, Cxcl5, Cxcl9* and *Cxcl10* was markedly increased, whereas the expression of the M2 surface marker *Cd206* was significantly decreased upon CCl_4_ treatment. However, lnc-Lfar1 silencing abrogated CCl_4_-induced dysregulation of these genes assessed by qRT-PCR (Fig. 2c). In addition, we performed immunohistochemistry-frozen, immunoblotting and qRT-PCR assays in the BDL-induced mice liver fibrosis model and obtained similar results (Supplementary Fig. 3a–c). Taken together, our data clearly reveal that lnc-Lfar1 silencing alleviates CCl_4_- and BDL-induced proinflammatory response of M1 macrophages.

### Silencing lnc-Lfar1 ameliorates CCl_4_- and BDL-induced NLRP3 inflammasome-mediated pyroptosis

**Fig. 3:**
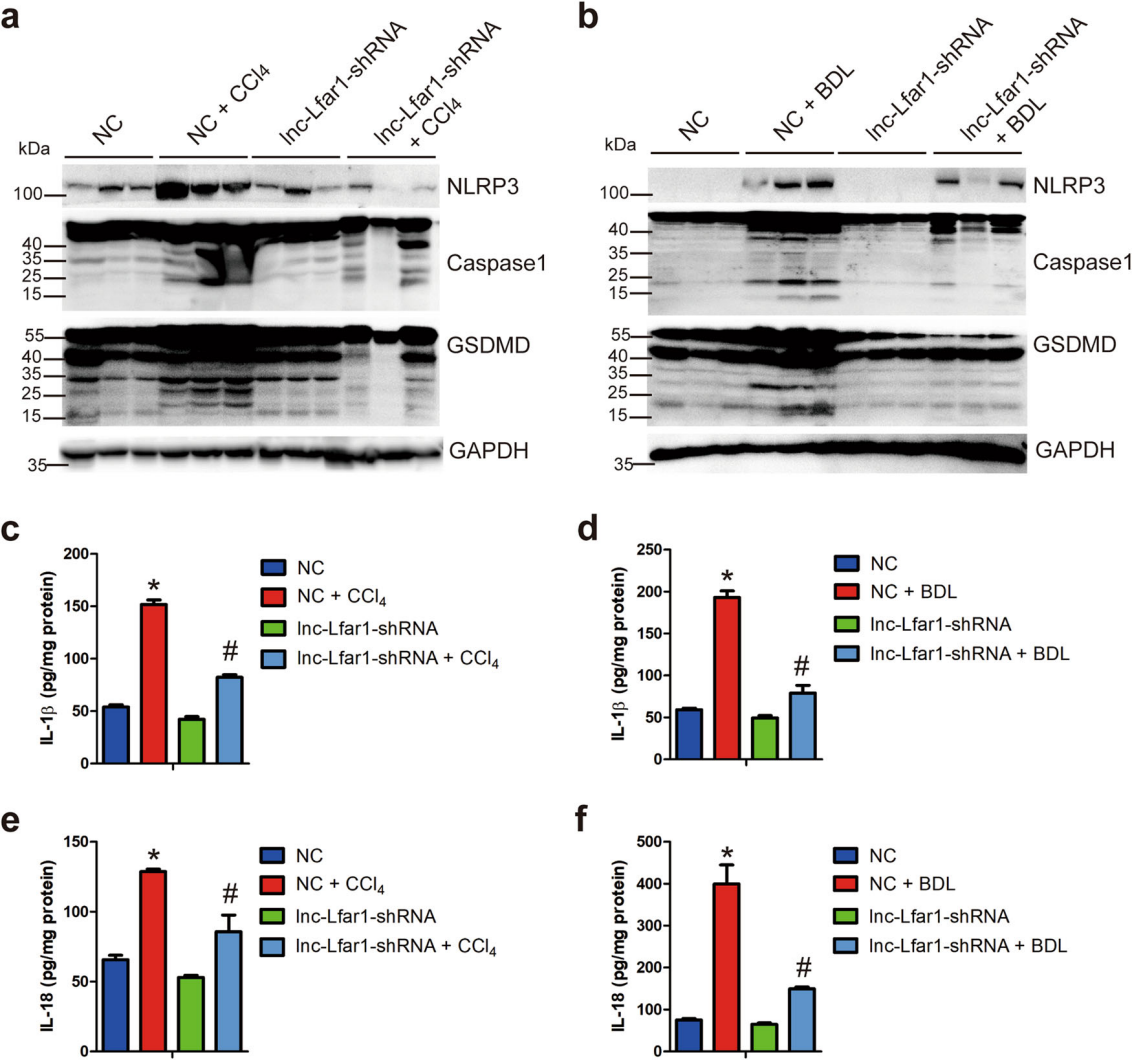
Silencing lnc-Lfar1 ameliorates CCl_4_- and BDL-induced NLRP3 inflammasome-mediated pyroptosis. Mice were treated with oil or sham operation in combination with injection of lenti-NC (NC), CCl_4_ or BDL operation in combination with injection of lenti-NC (NC + CCl_4_/BDL), oil or sham operation in combination with injection of lenti-lnc-Lfar1-shRNA (lnc-Lfar1-shRNA, *n* = 10), and CCl_4_ or BDL operation in combination with injection of lenti-lnc-Lfar1-shRNA (lnc-Lfar1-shRNA + CCl_4_ / BDL). **a**, **b** The protein level of NLRP3, Caspase1 and GSDMD was determined by western blot. GAPDH was used as an internal control. **c–f** The level of mature IL-1β and IL-18 in liver tissues was determined by ELISA. *p < 0.05 vs NC, ^#^*p* < 0.05 vs NC + CCl_4_/BDL.

Recent studies have shown that NLPR3 inflammasome and pyroptosis, which is a novel programmed and proinflammatory cell death, played a vital role in liver inflammation and fibrosis^2,3,10^. To determine the influence of lnc-Lfar1 on the pyroptosis during liver fibrosis process in vivo, we assessed pyroptosis by the level of key proteins regulating pyroptosis including NLRP3, pro-caspase-1, cleaved caspase1, GSDMD and GSDMD-N, mature IL-1β, IL-18 and lactate dehydrogenase (LDH) release. Immunoblotting analysis revealed that CCl_4_ and BDL treatment significantly increased the protein level of NLRP3, cleaved caspase1 and GSDMD-N, compared with the oil-treated group mice infected with NC, while lnc-Lfar1 silencing obviously decreased CCl_4_- and BDL-induced upregulation of these proteins (Fig. 3a, b). Moreover, the ELISA results demonstrated that the level of the mature IL-1β and IL-18 was markedly increased upon CCl_4_ and BDL treatment. However, downregulation of lnc-Lfar1 abrogated CCl_4_- and BDL-induced upregulation of these cytokines (Fig. 3c–f). In addition, lnc-Lfar1 knockdown attenuated CCl_4_- and BDL-induced release of LDH (Supplementary Fig. 4a, b). Taken together, these data demonstrate that lnc-Lfar1 silencing ameliorates CCl_4_- and BDL-induced NLRP3 inflammasome-mediated pyroptosis.

### Lnc-Lfar1 knockdown suppresses LPS- and IFN-γ-induced proinflammatory activation of macrophages

**Fig. 4:**
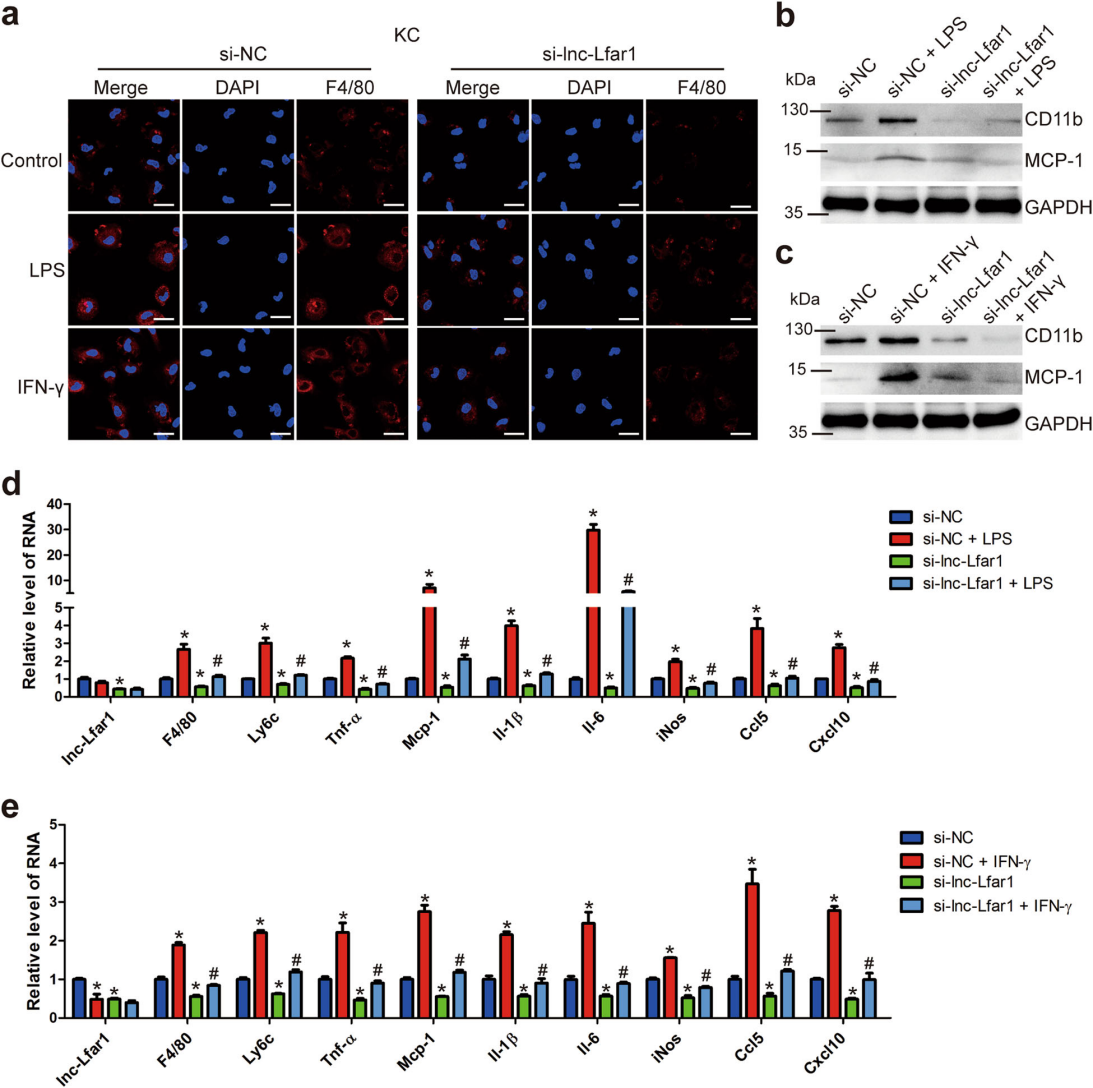
Lnc-Lfar1 knockdown suppresses LPS- and IFN-γ-induced proinflammatory activation of macrophages. KCs were transfected with lnc-Lfar1 siRNA and following treated with 20 ng/ml LPS or 100 ng/ml IFN-γ for 24 h. **a** The F4/80 expression was determined by confocal analysis; scale bar = 10 μm. **b**, **c** The protein level of CD11b and MCP-1 was determined by western blot. GAPDH was used as an internal control. **d**, **e** The RNA level of *lnc-Lfar1, F4/80, Ly6c*, *Tnf-α, Mcp-1, Il-1β, Il-6, iNos, Ccl5* and *Cxcl10* was determined by qRT-PCR. *p < 0.05 vs si-NC, ^#^*p* < 0.05 vs si-NC + LPS/IFN-γ.

We had shown that lnc-Lfar1 was required for the CCl_4_- and BDL-induced proinflammatory response of M1 macrophages in vivo. We then asked if lnc-Lfar1 has similar activity in vitro. Firstly, three different specific siRNAs targeting lnc-Lfar1 were used to assess the knockdown efficiency of lnc-Lfar1 in RAW264.7 cells. The results showed that all the three siRNAs decreased the expression of lnc-Lfar1, and the expression of *Tnf-α, Mcp-1, Il-1β* and *Il-6* was simultaneously down-regulated (Supplementary Fig. 5a). The si-lnc-Lfar1-3 with the better efficiency was selected for the subsequent experiments. Subsequently, mouse primary KCs were transfected with lnc-Lfar1 siRNA following a treatment with 20 ng/ml LPS or 100 ng/ml IFN-γ for 24 h. Confocal microscopy demonstrated that the expression of F4/80 was decreased when lnc-Lfar1 was knocked down, while the LPS- and IFN-γ-induced up-regulation of F4/80 was greatly blocked (Fig. 4a). Moreover, Immunoblotting analysis revealed that the expression of the CD11b and MCP-1 was increased upon LPS or IFN-γ treatment. However, knockdown of lnc-Lfar1 attenuated LPS- and IFN-γ-induced increase of these proteins (Fig. 4b, c). In addition, lnc-Lfar1 silencing decreased the mRNA level of the M1 surface markers *F4/80* and *Ly6c*, the proinflammatory genes including *Tnf-α, Mcp-1, Il-1β, Il-6, iNos, Ccl5* and *Cxcl10*, and abrogated LPS- and IFN-γ-induced upregulation of these genes assessed by qRT-PCR (Fig. 4d, e). Consistently, these findings were further confirmed in RAW264.7 cells (Supplementary Fig. 5b–e) and BMMs (Supplementary Fig. 6a–e). On the other hand, forced lnc-Lfar1 expression obviously up-regulated the expression of the M1 surface markers and the proinflammatory genes, and aggravated LPS- and IFN-γ-induced upregulation of these genes in mouse primary KCs (Supplementary Fig. 7a–d) and BMMs (Supplementary Fig. 8a–e). Taken together, these results demonstrate that lnc-Lfar1 silencing suppresses LPS- and IFN-γ-induced proinflammatory activation of macrophages.

### Lnc-Lfar1 knockdown inhibits LPS/ATP- and LPS/Nigericin-induced NLRP3 inflammasome-mediated pyroptosis

**Fig. 5:**
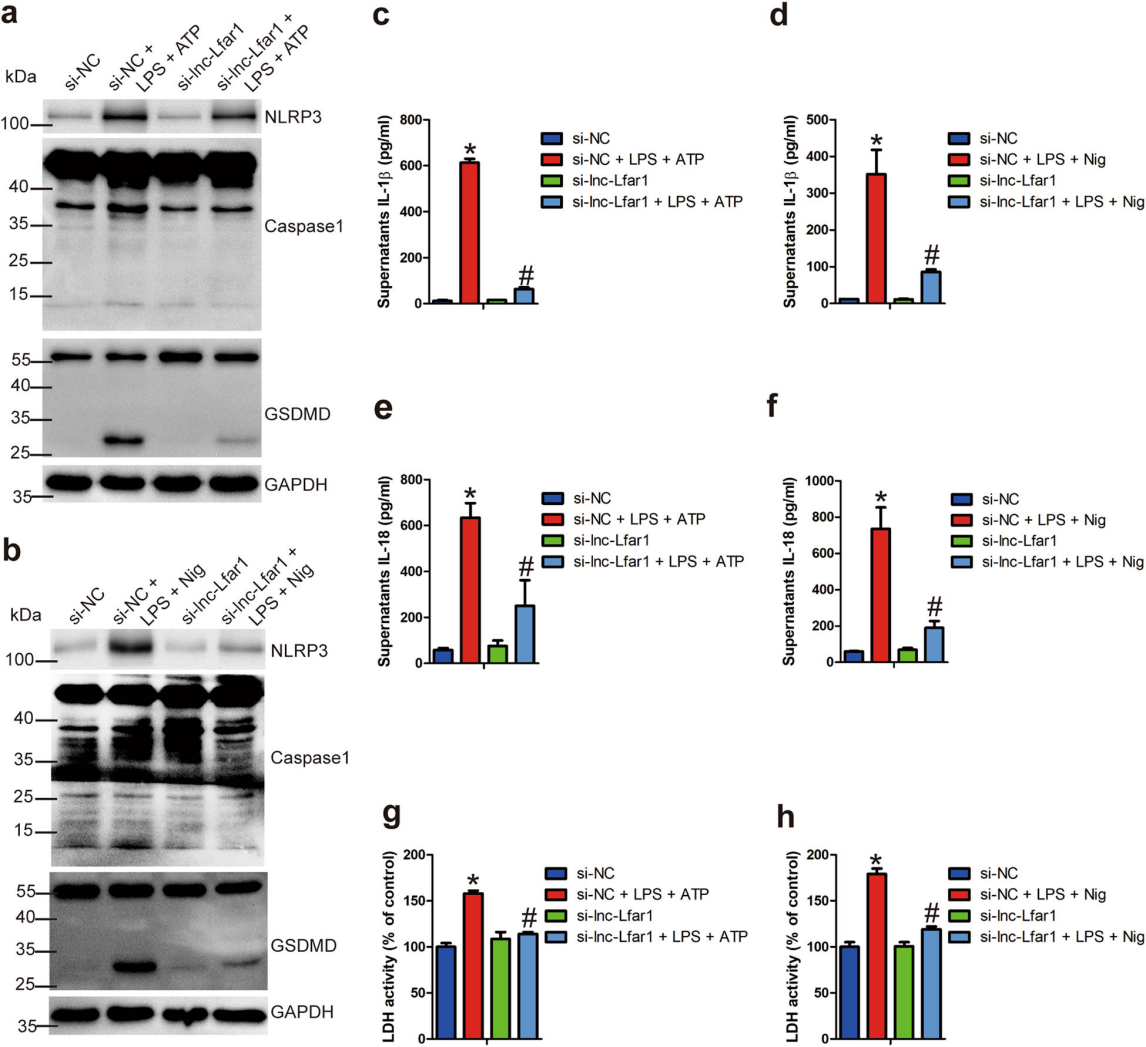
Lnc-Lfar1 knockdown inhibits LPS/ATP- and LPS/Nigericin-induced NLRP3 inflammasome-mediated pyroptosis. KCs were transfected with lnc-Lfar1 siRNA for 24 h, followed by stimulation with 100 ng/ml LPS for 4 h and subsequently treated with 5 mM ATP or 100 ng/ml for 2 h to induce pyroptosis. **a**, **b** The protein level of NLRP3, Caspase1 and GSDMD was determined by western blot. GAPDH was used as an internal control. **c–f** The level of mature IL-1β and IL-18 in the supernatant of cell culture medium was determined by ELISA. **g**, **h** Pyroptosis was measured by supernatant LDH activity. *p < 0.05 vs si-NC, ^#^*p* < 0.05 vs si-NC + LPS + ATP/Nig.

To further confirm the effects of lnc-Lfar1 on the pyroptosis from in vivo experiments, we transfected the KCs and BMMs with lnc-Lfar1 siRNA for 24 h or infected the cells with lenti-lnc-Lfar1 for 72 h, followed by inducing the NLRP3 inflammasome-mediated pyroptosis. qRT-PCR analysis suggested that LPS/ATP significantly decreased the expression of lnc-Lfar1 in KCs and BMMs, while LPS/Nigericin slightly down-regulated the level of lnc-Lfar1 in BMMs (Supplementary Fig. 9a–d). Moreover, the mRNA level of the pyroptosis-related genes *Nlrp3, pro-Caspase1, Asc, Il-1β, Il-18* and *Gsdmd* was increased upon LPS/ATP and LPS/Nigericin treatment. In addition, lnc-Lfar1 silencing abrogated, whereas forced lnc-Lfar1 expression promoted LPS/ATP- and LPS/Nigericin-induced dysregulation of these genes (Supplementary Fig. 9e–h; Supplementary Fig. 10a–d). The immunoblotting results also showed that LPS/ATP and LPS/Nigericin significantly up-regulated the protein level of NLRP3, cleaved caspase1 and GSDMD-N, while lnc-Lfar1 silencing obviously abrogated LPS/ATP- and LPS/Nigericin-induced upregulation of these proteins (Fig. 5a, b; Supplementary Fig. 11a, b). We also detected the level of mature IL-1β and IL-18 in the supernatant of cell culture medium by ELISA. As expected, the results revealed that the level of IL-1β and IL-18 was remarkably increased by LPS/ATP and LPS/Nigericin treatment but diminished by transfection with lnc-Lfar1 siRNA (Fig. 5c–f; Supplementary Fig. 11c–f). Likewise, lnc-Lfar1 knockdown attenuated LPS/ATP- and LPS/Nigericin-induced LDH release (Fig. 5g, h; Supplementary Fig. 11g, h). On the other hand, forced lnc-Lfar1 expression obviously aggravated LPS/ATP- and LPS/Nigericin-induced both the protein level of NLRP3, cleaved caspase1 and GSDMD-N, and the release of IL-1β, IL-18 and LDH in mouse primary KCs (Supplementary Fig. 12a–h) and BMMs (Supplementary Fig. 13a–h). Taken together, these data demonstrate that lnc-Lfar1 silencing ameliorates LPS/ATP- and LPS/Nigericin-induced NLRP3 inflammasome-mediated pyroptosis.

### Effect of lnc-Lfar1 on M1 activation and pyroptosis is NF-ĸB-dependent

**Fig. 6:**
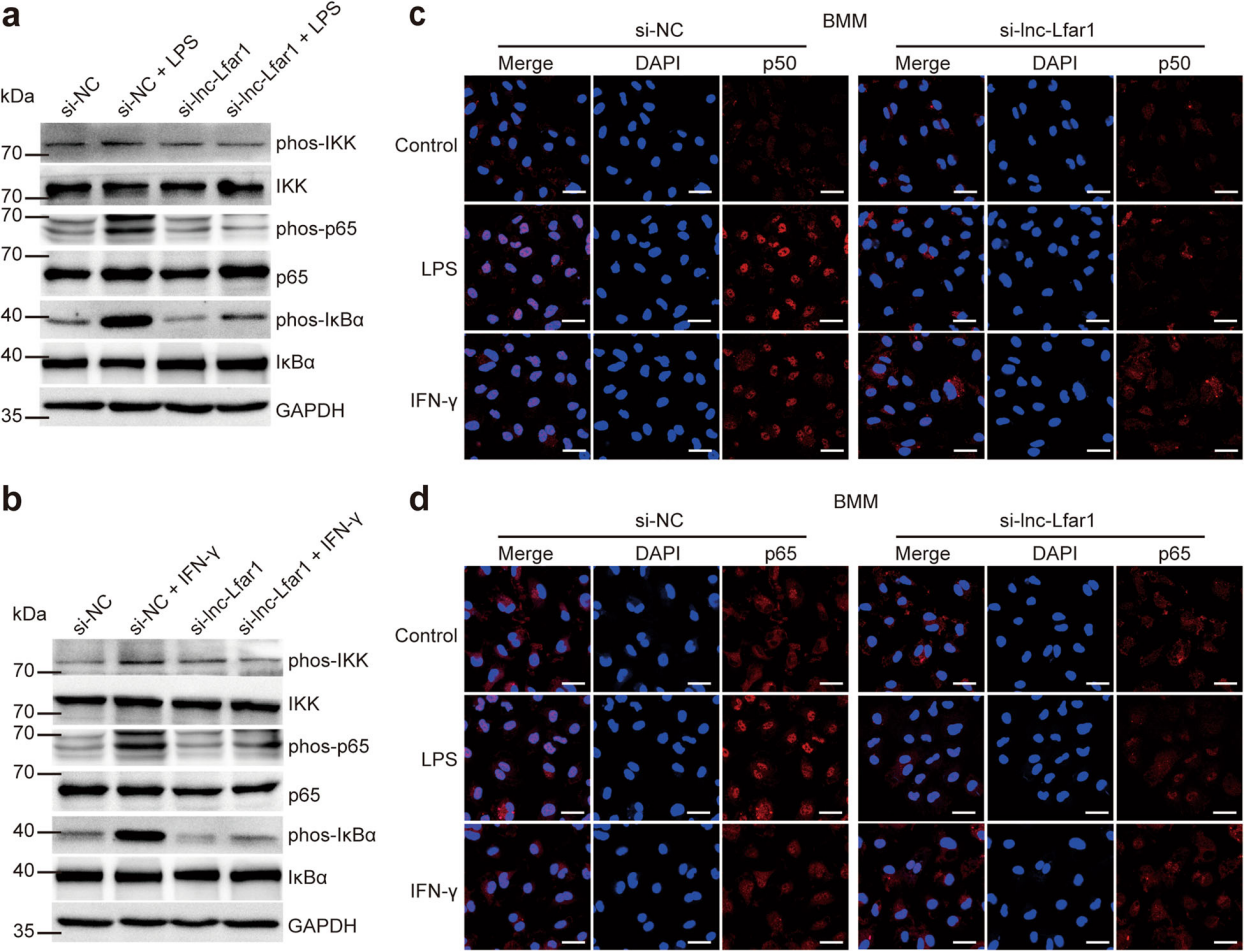
lnc-Lfar1 regulates macrophages activation through the NF-ĸB pathway. BMMs were transfected with lnc-Lfar1 siRNA and subsequently treated with 20 ng/ml LPS or 100 ng/ml IFN-γ for 24 h. **a**, **b** The protein level of phos-IKK, IKK, phos-p65, p65, phos-IĸBα and IĸBα was determined by western blot. GAPDH was used as an internal control. **c**, **d** The expression and location of p50 and p65 was determined by confocal analysis; scale bar = 10 μm.

Next, we investigated the mechanisms of lnc-Lfar1 on proinflammatory M1 macrophage activation and pyroptosis. Since NF-ĸB has been reported to be a critical signaling involved in inflammatory recruitment and liver fibrosis, and activation of NF-ĸB leads to constitutive overproduction of proinflammatory cytokines, including IL-1β, TNF-α, and MCP-1^20,21^, the effect of lnc-Lfar1 on NF-ĸB signaling was therefore investigated. BMMs were transfected with lnc-Lfar1 siRNA and subsequently treated with 100 ng/ml LPS or 20 ng/ml IFN-γ for 24 h. Immunoblotting analysis revealed that the phosphorylated- IKK, p65 and IĸBα, which is degraded and subsequently enables the NF-ĸB dimers to translocate into the nucleus to regulate gene expression, were markedly induced by LPS or IFN-γ treatment. However, knockdown of lnc-Lfar1 attenuated LPS- and IFN-γ-induced phosphorylation of IKK, p65 and IĸBα (Fig. 6a, b). Moreover, confocal microscopy demonstrated that LPS and IFN-γ promoted p50 and p65 to translocate into the nucleus, while lnc-Lfar1 silencing significantly attenuated LPS- and IFN-γ-induced p50 and p65 translocation (Fig. 6c, d). Consistently, these results were further confirmed in KCs and RAW264.7 cells (Supplementary Fig. 14a–d). On the other hand, forced lnc-Lfar1 expression aggravated LPS- and IFN-γ-induced phosphorylation of IKK, p65 and IĸBα, and promoted LPS- and IFN-γ-induced p50 and p65 translocation in BMMs (Supplementary Fig. 15a–d) and KCs (Supplementary Fig. 15e, f). Taken together, these results demonstrate that lnc-Lfar1 regulates LPS- and IFN-γ-induced proinflammatory activation of macrophages through the NF-ĸB pathway.

## Discussion

**Fig. 7:**
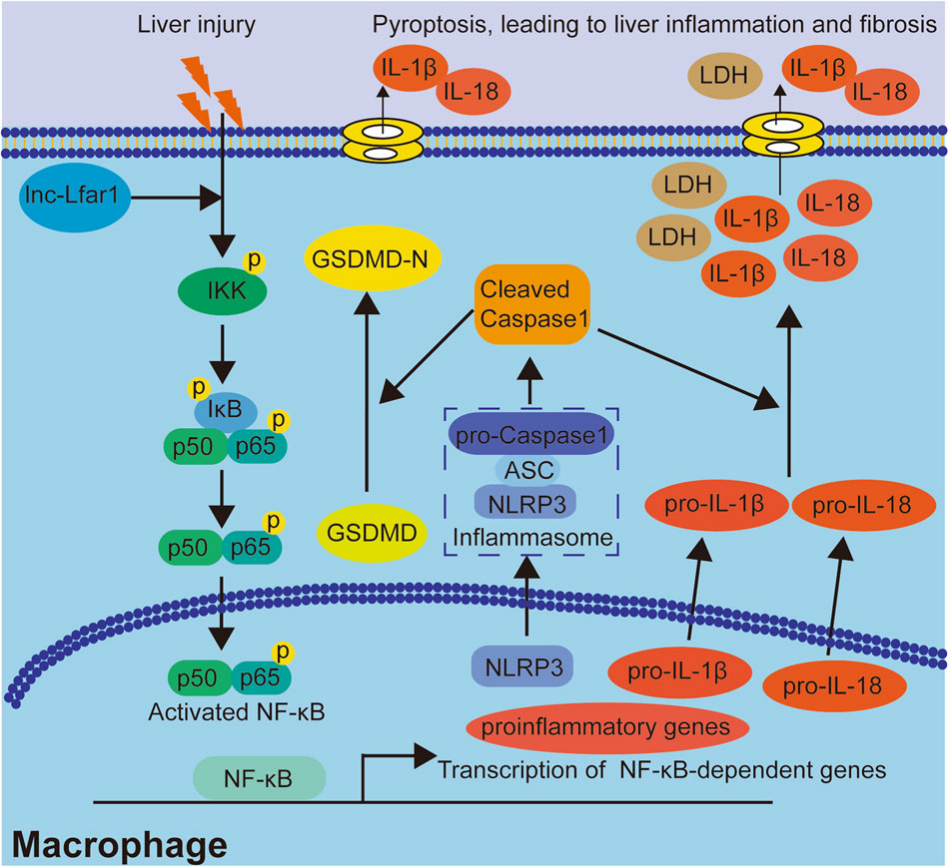
Schematic diagram illustrates the role of lnc-Lfar1 in the activation and pyroptosis of macrophage during liver fibrosis. In the proposed model, liver injury triggers NF-κB-mediated upregulation of NLRP3 and proinflammatory genes including IL-1β, IL-18, TNF-α, and MCP-1, which could be alleviated by lnc-Lfar1 silencing, thus providing an attractive therapeutic target against liver fibrosis.

Hepatic fibrosis is a common pathological consequence of a sustained wound healing response to persistent liver damage induced by various chronic liver diseases including hepatitis B and C, biliary obstruction, alcohol abuse, non-alcoholic steatohepatitis (NASH) and several other etiologies. If unresolved, the fibrotic process results in organ failure, and eventually death after the development of cirrhosis^1,2^. However, the molecular basis of hepatic fibrosis is incompletely understood, which has limited the identification of therapeutic targets. In this study, we provided evidence for a functional role of the liver-enriched lncRNA Lfar1 on the activation and pyroptosis of macrophage. We showed that lnc-Lfar1 silencing alleviated CCl_4_- and BDL-induced proinflammatory M1 macrophage activation and NLRP3 inflammasome-mediated pyroptosis in vivo, suppressed LPS- and IFN-γ-induced proinflammatory activation of macrophages, and inhibited LPS/ATP- and LPS/Nigericin-induced NLRP3 inflammasome-mediated pyroptosis in vitro. Mechanistically, lnc-Lfar1 promoted LPS- and IFN-γ-induced proinflammatory activation of macrophages through the NF-ĸB pathway (Fig. 7). All these data supported our conclusion that lnc-Lfar1 plays a vital role in controlling the activation and pyroptosis of macrophage, thus providing a possible therapeutic target against liver fibrosis.

It is well accepted that activated HSCs are the most principal cellular players promoting synthesis and deposition of ECM proteins in response to accumulated levels of inflammatory signals^1^. However, HMs are among the primary cells exposed to and responded to the liver injury, due to the anatomical location at the luminal side of the liver sinusoidal endothelium and their long cytoplasmic expansions^4,22^. It is now well recognized that HMs consists of ontogenically distinct populations termed KCs and MoMFs. KCs are self-renewing and liver-resident macrophages, exerting crucial functions during liver homeostasis, including clearance of systemic and intestine-derived pathogens^4^. When liver tissues are damaged during hepatitis B and C infection or following toxication, inflammasome is formed in KCs in response to PAMPs or DAMPs, triggering KCs activation. Subsequently, activated KCs release various cytokines and chemokines, such as IL-1β, IL-6, TNF-α, MCP-1, CCL5 and CXCL10, which perpetuate hepatic inflammation and recruit MoMFs into the liver. LY6C high-expressed MoMFs are recruited in a MCP-1-CCR2-dependent manner and secrete profibrotic cytokines including MCP-1, CCL5, TGFβ and PDGF, which promote the activation and survival of HSCs, the development of pathological fibrosis and can be pharmacologically inhibited by their corresponding antagonists^3–5,22,23^. In contrast, when the injury ceased, these profibrotic macrophages switch their phenotype towards antifibrotic macrophages that are characterized by the downregulation of LY6C and the enhanced expression of anti-inflammatory mediators, such as IL-10 and HGF, and matrix degrading metalloproteinases including MMP9, MMP12 and MMP13^4–6^. Thus, understanding of macrophage biology during liver fibrosis is a promising antifibrotic approach. In this study, we showed that lnc-Lfar1 silencing alleviated liver injury-induced upregulation of LY6C, IL-1β, IL-6, TNF-α, MCP-1, CCL5 and CXCL10 in vivo and in vitro, suggesting that lnc-Lfar1 knockdown might be beneficial for preventing liver fibrosis by targeting these chemokines. On the other hand, pyroptosis is a novel programmed and proinflammatory cell death, which is initiated by inflammasomes and executed by GSDMD, and plays a vital role in liver inflammation and fibrosis^2,7,8,11^. Therefore, inhibition of NLRP3 inflammasome and GSDMD could be a potential therapeutic approach for the prevention and therapy of liver inflammation and fibrosis through regulating pyroptosis. Of note, our data revealed that lnc-Lfar1 silencing inhibited pyroptosis in vivo and in vitro as assessed by the level of pyroptosis-related proteins including NLRP3, pro-caspase-1, cleaved caspase1, GSDMD and GSDMD-N, mature IL-1β, IL-18 and the released LDH, further confirming that lnc-Lfar1 may represent a therapeutic target for inflammasome associated diseases such as liver fibrosis.

It has been reported that up to 90% of eukaryotic genomes are transcribed, while the majority of transcripts are lncRNAs, which are a group of transcripts longer than 200 nucleotides but do not encode protein^12^. Despite their poor conservation and low levels of expression compared with protein-coding genes, more and more lncRNAs are reported to get involved in a variety of physiological and pathological processes, such as hepatic fibrosis^19,24–28^. For instance, lncRNA H19 aggravated cholestatic liver fibrosis either by promoting HSCs activation or by preventing ZEB1-mediated inhibition of epithelial cell adhesion molecule^25,29^. Yu et al.^27^ has demonstrated that lncRNA SNHG7 reduced miR-378a-3p and attenuated its control on DVL2, leading to aberrant Wnt/β-catenin activity that contributes to liver fibrotic progression. Our group has recently demonstrated that the nuclear-retained lncRNA SCARNA10 functioned as a novel positive regulator of TGFβ signaling in liver fibrogenesis by inhibiting the binding of PRC2 to the promoters of the genes associated with ECM and TGFβ pathway^28^. Moreover, we also reported that the liver-enriched lncRNA Lfar1 promoted HSCs activation and HC apoptosis, and subsequently leads to liver fibrosis^19^. In addition, lnc-Lfar1 showed a comparable expression level in HSCs and KCs, and the microarray analysis revealed that lnc-Lfar1 silencing affected a list of genes associated with immune response and responded to IFN-γ in CCl_4_-induced liver fibrosis. However, the effects of lnc-Lfar1 on macrophage biology during liver fibrosis are still unclear. In the present study, we demonstrated that lnc-Lfar1 promoted LPS- and IFN-γ-induced proinflammatory activation of macrophages, and subsequently induced proinflammation cytokines release during the progression of hepatic fibrogenesis. However, our data showed that lnc-Lfar1 expression is significantly reduced after CCL_4_ stimulation. This seems to be contradictory but could be a result of a negative feedback that lnc-Lfar1 expression is repressed when the proinflammatory M1 activation occurs. As mentioned above, based on the fact that lnc-Lfar1 promotes proinflammatory activation of macrophages, lnc-Lfar1 silencing leads to a further reduction of the target genes expression. The mechanism of action is reminiscent similarly to the expression and role of BMP9^30^, CXCL9^31^ and lnc-Lsm3b^32^ in hepatic fibrosis and innate immune response, respectively. Moreover, further studies are needed to investigate the interplay of lnc-Lfar1 with proinflammation cytokines and its possible mechanisms during hepatic fibrogenesis. On the other hand, recent reports have demonstrated that lncRNAs participate in modulating biological and pathological processes through regulating gene expression according to the cellular location^12^. In the cytoplasm, lncRNAs regulate gene expression by affecting the stability of mRNAs, altering the translation efficacy of target mRNAs and function as precursor of microRNAs or compete for microRNA-mediated inhibition, leading to increased expression of the mRNA. In the nucleus, lncRNAs control the epigenetic state of particular genes, participate in transcriptional regulation, involve in alternative splicing and constitute subnuclear compartments^12^. Since the lnc-Lfar1 locates both in the nucleus and cytoplasm of liver cells, to further investigate the molecular mechanism of lnc-Lfar1 on the nuclear translocation of p65 and p50, we also detected the transcription of the genes upstream the NF-ĸB pathway including Tlr4, Myd88, Ifnγr, Ikk1 and Ikk2, in lnc-Lfar1 downexpressed—and overexpressed—KCs or BMMs. The results showed that lnc-Lfar1 does not regulate these genes transcriptionally (Supplementary Fig. 16a−h), suggesting that lnc-Lfar1 may activate the NF-ĸB pathway post-transcriptionally.

In summary, our data identified lnc-Lfar1 plays a vital role in controlling the activation and pyroptosis of macrophage, thus providing an attractive therapeutic target against liver fibrosis.

## Materials and methods

### Antibodies and reagents

Antibodies used for western bloting included F4/80 (rat monoclonal, Abcam, ab16911; 1:200), CD11b (rabbit monoclonal, Abcam, ab133357; 1:1000), LY6C (rat monoclonal, Novus, NBP2-00441; 1:1000), MCP-1 (rabbit polyclonal, Cell Signaling Technology, #2029; 1:1000), NLRP3 (rabbit monoclonal, Cell Signaling Technology, #15101; 1:1000), pro-Caspase1 (rabbit monoclonal, Abcam, ab179515; 1:1000), GSDMD (rabbit monoclonal, Abcam, ab209845; 1:1000), IKK (rabbit monoclonal, Abcam, ab178870; 1:1000), phospho-IKK (rabbit monoclonal, Cell Signaling Technology, #2697; 1:1000), p65 (mouse monoclonal, Sabta cruz, sc-8008; 1:200), phospho-p65 (rabbit monoclonal, Cell Signaling Technology, #3033; 1:1000), IĸBα (mouse monoclonal, Cell Signaling Technology, #9246; 1:1000), phospho-IĸBα (mouse monoclonal, Cell Signaling Technology, #4814; 1:1000), GAPDH (mouse monoclonal, Abcam, ab8245; 1:8000), Goat Anti-Mouse IgG (Abcam, ab97023; 1:10000), Goat Anti-Rabbit IgG (Abcam, ab97051; 1:10000) and Goat Anti-Rat IgG (Abcam, ab97057; 1:10000). Antibodies used for immunohistochemistry and immunohistochemistry-frozen included F4/80 (rat monoclonal, Abcam, ab16911; 1:50) and LY6C (rat monoclonal, Novus, NBP2-00441; 1:200). LPS, IFN-γ, IL-4, IL-10 and M-CSF were purchased from PeproTech, ATP and nigericin were purchased from Med Chem Express.

### Cell culture and treatments

The murine immortalized macrophages RAW264.7 cells were maintained in Dulbecco’s modified Eagle’s medium (DMEM, Invitrogen, Camarillo, CA) supplemented with 10% fetal bovine serum (FBS, Gibco, Gaithersburg, MD, USA), penicillin (100 U/ml) and streptomycin (100 μg/ml). Bone marrow-derived macrophages (BMMs) were isolated from the femur and tibia of C57BL/6 mice (8–9 weeks) cultured in DMEM containing 10% FBS, penicillin (100 U/ml) and streptomycin (100 μg/ml) and 10 ng/mL murine M-CSF (Peprotech) for 6 days. Medium was changed every 2-day. Primary mouse KCs were isolated by pronase/collagenase perfusion digestion followed by subsequent density gradient centrifugation, as previously described. Magnetic cells sorting (MACS)-based positive selection using a F4/80 antibody (eBioscience) and or selective adhesion was further employed to purify KCs. RAW264.7 cells, BMMs and KCs were polarized into an M1 or M2 phenotype using 20 ng/ml INF-γ and 100 ng/ml LPS or 20 ng/ml IL-4, 20 ng/ml IL-10, respectively. Cells were primed with 100 ng/ml LPS for 4 h, and subsequently stimulated with 10 μM Nigericin or 5 mM ATP for 2 h to induce pyroptosis.

### Cell transfection

For gene knockdown analysis, siRNAs targeting the lnc-Lfar1 and and non-targeting siRNA (si-NC) were obtained from GenePharma Biological Technology (Shanghai, China). Cells were transfected with the siRNAs at 50% confluence using lipofectamine MAX according to the manufacturer’s instructions (Invitrogen, Grand Island, NY, USA). After culturing for 24–48 h, cells were analyzed by real-time PCR to determine knockdown efficiency. Target sequences of these siRNA were as follows: si-lnc-Lfar1-1: GGUCACGAUUCAUCUGAAATT; si-lnc-Lfar1-2: GGACCUCAUCUGUAAUGAATT; si-lnc-Lfar1-3: GAUCCUAUUAAACCGCUUATT; si-NC: GUUCUCCGAACGUGUCACGTT.

### Animal model and treatment

The CCl_4_- and BDL-induced mouse liver fibrosis model was previously described^19^. Animal protocols were approved by Tianjin Medical University Animal Care and Use Committee. The methods were carried out in accordance with the approved guidelines. All Balb/c mice aged at 8 weeks obtained from *Institute of Laboratory Animal Sciences, CAMS & PUMC* (Beijing, China).

### Immunohistochemistry

The specimens were sequentially fixed in 10% formalin for two days, transferred to ethanol of different concentration and embedded in paraffin in preparation for immunohistochemical analysis. Briefly, sections prepared on slides were first submitted to antigen retrieval by incubation in citrate buffer (pH 6.0) for 5 min at 108 °C and pretreated with 3% H_2_O_2_ for 15 min at room temperature followed by washing with PBS. Slides were subsequently incubated in normal goat serum for 20 min to block the nonspecific immunoreactivity. Next, the slides were treated with primary antibody overnight at 4 °C. The slides were incubated with secondary antibody (1:500) (HRP-conjugated anti-rabbit IgG). And the reaction products were visualized using diaminobenzidine (DAB) and monitored by microscopy.

### Confocal microscopy

Confocal immunofluorescent analysis was performed as described previously^19,28^. Antibodies used for confocal microscopy included F4/80 (rat monoclonal, Abcam, ab16911; 1:50), VEGFR2 (rabbit monoclonal, Cell Signaling Technology, #9698; 1:500), α-SMA (rabbit polyclonal, Abcam, ab5694; 1:100), p65(mouse monoclonal, Sabta cruz, sc-8008; 1:50), p50 (rabbit monoclonal, Cell Signaling Technology, #13586; 1:200) and goat anti rabbit/mouse/rat IgG (Life Technologies, Alexa Fluor 594; 1:500). All immunofluorescence was then visualized by a confocal microscope (LSM 700) or a fluorescence microscope.

### ELISA and LDH assay

Levels of IL-1β, IL-18 and LDH in cell cultured supernatants and liver tissue extracts were assessed using commercial assay kits (Nanjing Jiancheng, Nanjing, China) according to the manufacturer’s protocols.

### Western blot analysis

Immunoblotting analysis was performed as described previously^19,28^.

### Quantitative real-time polymerase chain reaction

Total RNA extracted from liver tissues or cells with Trizol reagent (Takara, Dalian, China). All RNA was digested with DNase I (Takara, Dalian, China). For real-time PCR, all reactions were performed in triplicate with SYBR Green master mix (Takara, Dalian, China) according to the manufacturer’s instructions. The expression level of housekeeping gene GAPDH was used to normalize the expression level of the genes-of-interest. Primers sequences for mouse genes were Gapdh sense 5′-GGCATGGACTGTGGTCATGAG-3′ and antisense 5′-TGCACCACCAA-CTGCTTAGC-3′; lnc-Lfar1 sense 5′-GCCAGCACACTAAAGACGAG-3′ and antisense 5′-GCAAAGGTGGAGGTCAGATT-3′; Ly6C sense 5′-GCAGTGCTACGAGTGCTATGG-3′ and antisense 5′-ACTGACGGGTCTTTAGTTTCCTT-3′; F4/80 sense 5′-TGACTCACCTTGTGGTC-CTAA-3′ and antisense 5′-CTTCCCAGAATCCAGTCTTTCC-3′; Ccr2 sense 5′-ATGCAAG-TTCAGCTGCCTGC-3′ and antisense 5′-ATGCCGTGGATGAACTGAGG-3′; Cd206 sense 5′-GTGGAGTGATGGAACCCCAG-3′ and antisense 5′-CTGTCCGCCCAGTATCCATC-3′; Arg1 sense 5′-ACATTGGCTTGCGAGACGTA-3′ and antisense 5′-ATCACCTTGCCAATCCCCAG-3′; Tnf-α sense 5′-CATCTTCTCAAAATTCGAGTGACAA-3′ and antisense 5′-TGGGAGTAGACA-AGGTACAACCC-3′; Il-1β sense 5′-GTCGCTCAGGGTCACAAGAA-3′ and antisense 5′-GTG-CTGCCTAATGTCCCCTT-3′; Mcp-1 sense 5′-GTTAACGCCCCACTCACCTG-3′ and antisense 5′-GGGCCGGGGTATGTAACTCA-3′; iNos sense 5′-CAGGGCCACCTCTACATTTG-3′ and antisense 5′- TGCCCCATAGGAAAAGACTG -3′; Il-6 sense 5′-AGTTGCCTTCTTGGGACTGA-3′ and antisense 5′-TCCACGATTTCCCAGAGAAC-3′; Ccl5 sense 5′-CCACTTCTTCTCTGGG-TTGG-3′ and antisense 5′-GTGCCCACGTCAAGGAGTAT-3′; Cxcl5 sense 5′-TGCATTCCGCTTAGCTTTCT-3′ and antisense 5′-CAGAAGGAGGTCTGTCTGGA-3′; Cxcl9 sense 5′-TCTTGGGCATCATCTTCCTGG-3′ and antisense 5′-GAGGTCTTTGAGGGATTTGT-AGTGG-3′; Cxcl10 sense 5′-GATGACGGGCCAGTGAGAAT-3′ and antisense 5′-CTCAACA-CGTGGGCAGGATA-3′; Nlrp3 sense 5′-CGGATGTTATTCTGGCAACA-3′ and antisense 5′-CGCTTTGGAGATGGATCTGT-3′; Pro-Caspase1 sense 5′-AGATGGCACATTTCCAGGAC-3′ and antisense 5′- GATCCTCCAGCAGCAACTTC -3′; Asc sense 5′-TGACAGTGCAACTGCGA-GAA-3′ and antisense 5′-GTGAGCTCCAAGCCATACGA-3′; Il-18 sense 5′-CACTTCTC-CCCTGTGGTGTG-3′ and antisense 5′-GGCAGGAGTCCAGAAAGCAT-3′; Gsdmd sense 5′-GCTCTATGCCTCCCTGTTCC-3′ and antisense 5′-TTCTACCTTGGCTGGAGGGT-3′; Tlr4 sense 5′-TTTGCTGGGGCTCATTCACT-3′ and antisense 5′-GACTCGGCACTTAGCACTGT-3′; Myd88 sense 5′-CTCGCAGTTTGTTGGATGCC-3′ and antisense 5′-GGCCACCTGTAAA-GGCTTCT-3′; Ikk1 sense 5′-GTTCTGCCCGCTCTCTTGTA-3′ and antisense 5′- TCACACA-TGTCAGAGGATGTTCA-3′; Ikk2 sense 5′-GTGCCTGTGACAGCTTACCT-3′ and antisense 5′-ACTGCGTTTGCACTTTTGCT-3′; Ifnγr1 sense 5′-ACGGTGATCTGTGAAGAGCC-3′ and antisense 5′-TTTGTGTCGGAGTTGGAGGG-3′.

### Statistical analysis

Data were expressed as mean ± SD. All the statistical analyses were performed with the SPSS 13.0 (IBM, Armonk, NY, USA). Statistical analyses were performed using either Student’s *t*-test (two-group comparison) or one-way analysis of variance (more than two groups) followed by post hoc comparison, and differences with *p* < 0.05 were considered significantly.

## Supplementary information

Supplementary Figure Legends

Supplementary Figure 1

Supplementary Figure 2

Supplementary Figure 3

Supplementary Figure 4

Supplementary Figure 5

Supplementary Figure 6

Supplementary Figure 7

Supplementary Figure 8

Supplementary Figure 9

Supplementary Figure 10

Supplementary Figure 11

Supplementary Figure 12

Supplementary Figure 13

Supplementary Figure 14

Supplementary Figure 15

Supplementary Figure 16
