# Supplementary material for: Silencing lncRNA Lfar1 alleviates the classical activation and pyoptosis of macrophage in hepatic fibrosis
Source: Cell Death Dis. 2020 Feb 18;11(2):132. doi: 10.1038/s41419-020-2323-5 (PMC7028920; doi:10.1038/s41419-020-2323-5)
Supplement: Supplementary file 1 — Supplementary Figure Legends [file 41419_2020_2323_MOESM1_ESM.docx]

**Supplementary Figure S1, related to Fig. 1**

KCs were identified by confocal analysis for F4/80, α-SMA and VEGFR2

**Supplementary Figure S2, related to Fig. 1**

**a-f** KCs, RAW264.7 cells and BMMs were stimulated with 20 ng/ml IL-4 or 20 ng/ml IL-10 for 24 h and the expression of *lnc-Lfar1* and positive control genes (*Arg1* or *Cd206*) was determined by qRT-PCR. **p* < 0.05 vs control.

**Supplementary Figure S3, related to Fig. 2 Silencing lnc-Lfar1 alleviates BDL-induced** **proinﬂammatory activation of macrophages.**

Mice were treated with sham operation in combination with injection of lenti-NC (NC, n=15), BDL operation in combination with injection of lenti-NC (NC + BDL, n=15), sham operation in combination with injection of lenti- lnc-Lfar1-shRNA (shLFAR1, n=15) and BDL operation in combination with injection of lenti- lnc-Lfar1-shRNA (lnc-Lfar1-shRNA + BDL, n=15). **a** Immunohistochemistry-frozen analysis wa performed to detect the expression of F4/80 and LY6C; scale bar = 100 μm. **b** The protein level of F4/80, CD11b and LY6C was determined by western blot. GAPDH was used as an internal control. **c** The RNA level of *lnc-Lfar1, F4/80, Ly6c*, *Ccr2*, *Cd20,* *Il-6, iNos, Ccl5, Cxcl5, Cxcl9* and *Cxcl10* was determined by qRT-PCR. **p*<0.05 vs NC, #*p*<0.05 vs NC + BDL.

**Supplementary Figure S4, related to Fig. 3**

Mice were treated with oil or sham operation in combination with injection of lenti-NC (NC), CCl_4_ or BDL operation in combination with injection of lenti-NC (NC+CCl_4_ / BDL), oil or sham operation in combination with injection of lenti-lnc-Lfar1-shRNA (lnc-Lfar1-shRNA, n=10), and CCl_4_ or BDL operation in combination with injection of lenti-lnc-Lfar1-shRNA (lnc-Lfar1-shRNA +CCl_4_ / BDL). **a, b** Pyroptosis was measured by liver tissues LDH activity. **p*<0.05 vs NC, #*p*<0.05 vs NC + CCl_4_ / BDL.

**Supplementary Figure S5, related to Fig. 4**

**a** RAW264.7 cells were transfected with three different specific siRNAs respectively, the expression of *lnc-Lfar1*, *Tnf-α, Mcp-1, Il-1β* and *Il-6* was determined by qRT-PCR. **b-e** RAW264.7 cells were transfected with lnc-Lfar1 siRNA and following treated with 20 ng/ml LPS or 100ng/ml IFN-γ for 24 hours. The protein level of CD11b and MCP-1 was determined by western blot. GAPDH was used as an internal control (**b, c**). The RNA level of *lnc-Lfar1, F4/80, Ly6c*, *Tnf-α, Mcp-1, Il-1β, Il-6, iNos, Ccl5* and *Cxcl10* was determined by qRT-PCR (**d, e**). **p*<0.05 vs si-NC, #*p*<0.05 vs si-NC + LPS / IFN-γ.

**Supplementary Figure S6, related to Fig. 4**

BMMs were transfected with lnc-Lfar1 siRNA and following treated with 20 ng/ml LPS or 100ng/ml IFN-γ for 24 hours. **a** The F4/80 expression was determined by confocal analysis; scale bar = 10 μm. **b, c** The protein level of CD11b and MCP-1 was determined by western blot. GAPDH was used as an internal control. **d, e** The RNA level of *lnc-Lfar1, F4/80, Ly6c*, *Tnf-α, Mcp-1, Il-1β, Il-6, iNos, Ccl5* and *Cxcl10* was determined by qRT-PCR. **p*<0.05 vs si-NC, #*p*<0.05 vs si-NC + LPS / IFN-γ.

**Supplementary Figure S7, related to Fig. 4**

KCs were infected with lenti-lnc-Lfar1 for 48 hours and following treated with 20 ng/ml LPS or 100ng/ml IFN-γ for 24 hours. **a, b** The protein level of CD11b and MCP-1 was determined by western blot. GAPDH was used as an internal control. **c, d** The RNA level of *lnc-Lfar1, F4/80, Ly6c*, *Tnf-α, Mcp-1, Il-1β, Il-6, iNos, Ccl5* and *Cxcl10* was determined by qRT-PCR. **p*<0.05 vs LV-Control, #*p*<0.05 vs LV-Control + LPS / IFN-γ.

**Supplementary Figure S8, related to Fig. 4**

BMMs were infected with lenti-lnc-Lfar1 for 48 hours and following treated with 20 ng/ml LPS or 100ng/ml IFN-γ for 24 hours. **a** The F4/80 expression was determined by confocal analysis; scale bar = 10 μm. **b, c** The protein level of CD11b and MCP-1 was determined by western blot. GAPDH was used as an internal control. **d, e** The RNA level of *lnc-Lfar1, F4/80, Ly6c*, *Tnf-α, Mcp-1, Il-1β, Il-6, iNos, Ccl5* and *Cxcl10* was determined by qRT-PCR. **p*<0.05 vs LV-Control, #*p*<0.05 vs LV-Control + LPS / IFN-γ.

**Supplementary Figure S9, related to Fig. 5**

**a-d** KCs and BMMs were stimulated with 100 ng/ml LPS for 4 hours and subsequently treated them with 5 mM ATP or 100ng/ml for 2 hours to induce pyroptosis. The RNA level of *lnc-Lfar1* was determined by qRT-PCR. **p*<0.05 vs control. **e-h** KCs and BMMs were transfected with lnc-Lfar1 siRNA for 24 hours, then these cells were stimulated with 100 ng/ml LPS for 4 hours and subsequently treated them with 5 mM ATP or 100ng/ml for 2 hours to induce pyroptosis. The RNA level of *lnc-Lfar1, Nlrp3, pro-Caspase1, Asc, Il-1β, Il-18* and *Gsdmd* was determined by qRT-PCR. **p*<0.05 vs si-NC, #*p*<0.05 vs si-NC + LPS + ATP / Nig.

**Supplementary Figure S10, related to Fig. 5**

**a-d** KCs and BMMs were infected with lenti-lnc-Lfar1 for 72 hours, then these cells were stimulated with 100 ng/ml LPS for 4 hours and subsequently treated them with 5 mM ATP or 100ng/ml for 2 hours to induce pyroptosis. The RNA level of *lnc-Lfar1, Nlrp3, pro-Caspase1, Asc, Il-1β, Il-18* and *Gsdmd* was determined by qRT-PCR. **p*<0.05 vs LV-Control, #*p*<0.05 vs LV-Control + LPS + ATP / Nig.

**Supplementary Figure S11, related to Fig. 5**

BMMs were transfected with lnc-Lfar1 siRNA for 24 hours, then these cells were stimulated with 100 ng/ml LPS for 4 hours and subsequently treated them with 5 mM ATP or 100ng/ml for 2 hours to induce pyroptosis. **a, b** The protein level of NLRP3, Caspase1 and GSDMD was determined by western blot. GAPDH was used as an internal control. **c-f** The level of mature IL-1β and IL-18 in the supernatant of cell culture medium was determined by ELISA. **g, h** Pyroptosis was measured by supernatant LDH activity. **p*<0.05 vs si-NC, #*p*<0.05 vs si-NC + LPS + ATP / Nig.

**Supplementary Figure S12, related to Fig. 5**

KCs were infected with lenti-lnc-Lfar1 for 72 hours, then these cells were stimulated with 100 ng/ml LPS for 4 hours and subsequently treated them with 5 mM ATP or 100ng/ml for 2 hours to induce pyroptosis. **a, b** The protein level of NLRP3, Caspase1 and GSDMD was determined by western blot. GAPDH was used as an internal control. **c-f** The level of mature IL-1β and IL-18 in the supernatant of cell culture medium was determined by ELISA. **g, h** Pyroptosis was measured by supernatant LDH activity. **p*<0.05 vs LV-Control, #*p*<0.05 vs LV-Control + LPS + ATP / Nig.

**Supplementary Figure S13, related to Fig. 5**

BMMs were infected with lenti-lnc-Lfar1 for 72 hours, then these cells were stimulated with 100 ng/ml LPS for 4 hours and subsequently treated them with 5 mM ATP or 100ng/ml for 2 hours to induce pyroptosis. **a, b** The protein level of NLRP3, Caspase1 and GSDMD was determined by western blot. GAPDH was used as an internal control. **c-f** The level of mature IL-1β and IL-18 in the supernatant of cell culture medium was determined by ELISA. **g, h** Pyroptosis was measured by supernatant LDH activity. **p*<0.05 vs LV-Control, #*p*<0.05 vs LV-Control + LPS + ATP / Nig.

**Supplementary Figure S14, related to Fig. 6**

**a-d** KCs (**a, b**) and RAW264.7 cells (**c, d**) were transfected with lnc-Lfar1 siRNA and following treated with 20 ng/ml LPS or 100ng/ml IFN-γ for 24 hours. The protein level of phos-IKK, IKK, phos-p65, p65, phos-IĸBα and IĸBα was determined by western blot. GAPDH was used as an internal control.

**Supplementary Figure S15, related to Fig. 6**

BMMs were infected with lenti-lnc-Lfar1 for 48 hours and following treated with 20 ng/ml LPS or 100ng/ml IFN-γ for 24 hours. **a, b** The protein level of phos-IKK, IKK, phos-p65, p65, phos-IĸBα and IĸBα was determined by western blot. **c, d** The expression and location of p50 and p65 was determined by confocal analysis; scale bar = 10 μm. **e, f** KCs were infected with lenti-lnc-Lfar1 for 48 hours and following treated with 20 ng/ml LPS or 100ng/ml IFN-γ for 24 hours. The protein level of phos-IKK, IKK, phos-p65, p65, phos-IĸBα and IĸBα was determined by western blot. GAPDH was used as an internal control.

**Supplementary Figure S16, related to Fig. 6**

**a-d** KCs (**a, b**) and BMMs (**c, d**) were transfected with lnc-Lfar1 siRNA and following treated with 20 ng/ml LPS or 100ng/ml IFN-γ for 24 hours. The mRNA level of *Tlr4, Myd88, Ifnγr, Ikk1* and *Ikk2* was determined by qRT-PCR. **p*<0.05 vs si-NC, #*p*<0.05 vs si-NC + LPS / IFN-γ. **e-h** KCs (**e, f**) and BMMs (**g, h**) were infected with lenti-lnc-Lfar1 for 48 hours and following treated with 20 ng/ml LPS or 100ng/ml IFN-γ for 24 hours. The mRNA level of *Tlr4, Myd88, Ifnγr, Ikk1* and *Ikk2* was determined by qRT-PCR. **p*<0.05 vs LV-Control, #*p*<0.05 vs LV-Control + LPS / IFN-γ.
